# Fighting Science with Science: Counter-Expertise Production in Anti-Shale Gas Mobilizations in France and Poland

**DOI:** 10.1007/s00048-022-00342-x

**Published:** 2022-08-04

**Authors:** Roberto Cantoni

**Affiliations:** 1 Science Policy Research Unit (SPRU), University of Sussex, Jubilee Building G08, Falmer, BN1 9SL Brighton, UK; 2 LATTS-Ecole des Ponts ParisTech, 6 et 8, avenue Blaise Pascal, 77455 Marne-la-Vallée, France; 3 Dipartimento di Scienze Sociali, Università di Napoli ’Federico II’, Vico Monte della Pietà 1, 80138 Naples, Italy

**Keywords:** Shale gas, Poland, France, Mobilizations, Expertise, Schiefergas, Polen, Frankreich, Mobilisierung, Expertise

## Abstract

Between the second half of the 2000s and the first half of the 2010s, the prospect of shale gas extraction in Europe at first prompted fervent political support, then met with local and national opposition, and was finally rendered moot by a global collapse in the oil price. In the Europe-wide protests against shale gas and the main technique employed to extract it, hydraulic fracturing (or *fracking*), counter-expertise played a crucial role. This kind of expertise is one of the main elements of “energy citizenship,” a concept recently developed in the field of energy humanities which describes the empowerment of citizens in decision-making processes related to energy issues. This paper provides a socio-historical analysis of the co-production of counter-expertise and energy citizenship in the two European countries endowed with the largest shale gas reserves: Poland and France. In my analysis, concerns over the disruption of the food-water-energy nexus due to possible pollution emerge as particularly important. I argue that the exchange of information between activist groups and NGOs, as well as between activist groups from distant European locations, allowed for the creation of a genuinely transnational and science-based anti-fracking movement.

## Zuammenfassung

Zwischen der zweiten Hälfte der 2000er und der ersten Hälfte der 2010er Jahre, löste die Aussicht auf die Förderung von Schiefergas in Europa zuerst weitreichende politische Begeisterung aus, dann Gegeninitiativen auf der lokalen und nationalen Ebene, und schließlich Resignation, als ein weltweiter Einbruch des Ölpreises die Gewinnannahmen des Unternehmens in Frage stellte. Im Erfolg der europäischen Proteste gegen Schiefergas und „hydraulic fracturing“ (oder Fracking) war die Rolle der Gegenexpertise entscheidend. Diese Art von Expertise ist eines der Hauptelemente von „energy citizenship“, ein Konzept das kürzlich im Bereich der „*Energy Humanities*“ entwickelt wurde und für die Befähigung des Bürgers zur Entscheidungsfindung in energiebezogenen Fragen steht. *Die vorliegende Studie* analysiert die kooperative Produktion von Gegenexpertise und die *Schaffung* von „energy citizenship“ in den beiden europäischen Ländern, die mutmaßlich über die größten Schiefergasvorkommen verfügen: Polen und Frankreich. Aus dieser Analyse geht hervor, dass Besorgnis über die Störung des Nahrungsmittel-Wasser-Energie-Nexus aufgrund einer möglichen Verschmutzung durch industrielle Aktivitäten im Vordergrund stand. Der Informationsaustausch zwischen Aktivistengruppen und spezialisierten NGOs sowie zwischen Aktivistengruppen *in verschiedenen europäischen Orten *ermöglichte die Entstehung einer transnationalen, wissenschaftsbasierten Anti-Fracking-Bewegung.

## The Swift Rise and Fall of Europe’s Shale Gas Dreams

While shale gas extraction in Europe was never more than a theoretical hope, the continent’s love-hate relationship with it is one of the most compelling stories connecting civil society to energy production over the last 15 years. It contains all the elements of a good book: an exciting event, protagonists and antagonists, conflict, and resolution. The event was a report published by the US Energy Information Administration (EIA) in 2011 (EIA 2011) describing the geological potential of certain areas of the globe in terms of shale gas and oil resources. The protagonist was gas trapped deep underground, in rocks known as shale. The protagonist’s helpers were the oil and gas industry, as well as policymakers across virtually the entire ideological spectrum. The antagonists were the anti-shale movements that mushroomed in Europe wherever shale gas extraction was planned. The conflict was that between shale gas advocates and shale gas opponents, and its resolution was the ultimate death of the shale gas epic by a mixture of causes: an unexpected drop in oil and gas prices, which made further investments uneconomical; political anxiety stemming from anti-shale protests; and, in some cases, geological hurdles that made extraction unprofitable. The one element missing from this story is the key that would set shale gas free from its geological dungeon: a technique known as hydraulic fracturing, or *fracking*, which involves the fracturing of bedrock formations by a pressurized liquid (usually a mix of water, sand, and chemicals). Combined with a second technique called horizontal drilling, fracking would allow for the extraction of shale gas.

In fact, however, the EIA’s 2011 report is not really the beginning of the story. Since roughly 2007, the application of this combination of technologies in the United States has been resulted in a dramatic increase in gas production, transforming the US into world’s largest gas producer. Following the publication of several studies on the potential reserves of shale gas in specific countries (including the EIA’s report), European governments began to show interest in extracting this resource. Among the first to do so were the Polish and French governments, Poland and France being the countries with the largest continental reserves, according to the EIA report (5.3 and 5.1 trillion cubic meters, respectively). In the beginning of the 2010s, initiatives aimed at exploring and extracting this hydrocarbon spread over the continent, ultimately leading to disappointing results. By 2015, Europe’s shale gas dream was over. It had lasted less than ten years.

Because of the North American origins of fracking and its early application there, it is not surprising that the first political mobilizations against shale gas took place in the United States, especially in the most densely populated areas where fracking was used. The US was also the first country to see a documentary film critical of shale gas extraction produced. *Gasland*, directed by Josh Fox, was released in 2010 and quickly became an important reference point for mobilizations across the Atlantic. In Europe, the anti-shale mobilization was certainly not a first in technoscience-related protests. Earlier examples include movements against nuclear power, with mass opposition starting in the 1960s, and later, in the 1990s, against GMOs. It was, however, the first time that oil and gas had become the object of a transnational, environmental, and scientific controversy in Europe.

This paper focuses on the technology of fracking as it was rendered in the narratives produced by anti-shale movements. By analyzing their approaches to the production of scientific knowledge, it explores the motivations that formed the basis of their opposition to shale. It also challenges a binary view which juxtaposes “official” and “lay” experts, too often neglecting a range of “in-between” figures of non-official, or no-longer-official, experts. In the cases discussed here, the role of these outsiders, or “maverick experts,” was crucial.

Not surprisingly, anti-shale mobilizations developed with varying intensity in different European countries, depending on the energy context specific to each, and this contextual element had a considerable weight in each mobilization’s origins, actions, and outcomes. For example, the two countries that I have chosen as objects of study, France and Poland, are characterized by major historical differences in terms of public protest traditions. As noted by the environmental sociologist Christopher Rootes, while an independent and pluralistic environmental movement did emerge in Poland in the 1980s, “[i]t was not an active participant in the new, post-communist political institutions” (1997: 335). In addition, at the time of the controversy, the level of environmental awareness was still lower in Poland than in other EU countries (Szostek 2012: 259). By contrast, ever since the 1970s, and mainly coinciding with the expansion of the nuclear industry, the French population has shown a noteworthy ability to mobilize around technoscientific issues (Hayes 2006).

I focus on France and Poland primarily because of how differently their respective governments responded to the prospect of shale gas extraction. My choice was also a consequence of these two countries being attributed Europe’s largest shale gas resources in the EIA’s 2011 report. Aside from highlighting the social, historical, and political differences between France and Poland, leading to their respective anti-fracking mobilizations, this comparison also allows me to analyze the different pathways they took in the production of non-official expertise. The high level of institutionalization among environmental organizations in France allowed for a more structured production there, with multiple origins within France and with conflicts between different groups. On the contrary, in Poland, on account of the much smaller mobilization and the more recent institutionalization of environmental movements, transnational exchanges of information played a larger role.

These differences led to different outcomes. In France, any ambition on the part of the industry for a rapid and effective exploration of national resources was abandoned following the passage of Law n. 2011-835 in July 2011 (the so-called “Jacob Law”), prohibiting fracking. In Poland, by contrast, the green light given to the industry by the government enabled the continuation of exploratory activities. These, however, ultimately resulted in unprofitable prospects for extraction. Foreign companies started to withdraw from their Polish concessions as early as 2012, and by the end of 2015, most of them had left the country (Table 2), burdening national companies with continuing their research with reduced means.

This paper is structured as follows. In the next section, I will summarize the current state of socio-historical research on anti-shale mobilizations in Europe and provide a methodological note. I will then turn to my two case studies and analyze their origins, trajectories, and outcomes. In doing so, I will emphasize the dynamics of counter-expertise formation and transnational collaboration. I will use the paper’s conclusion to reflect on the issue of energy citizenship and on the democratization of scientific activity more broadly.

## Filling the Expertise Gap in a Wide-Ranging Literature

Academic interest in shale gas and fracking began in the mid-2010s, when researchers from a variety of disciplines—from psychology to sociology and from anthropology to political science—began to analyze phenomena related to this particular mode of resource extraction. The field of science and technology studies (STS), to which this study belongs, has been part of this trend (Cantoni et al. 2018a; Lis and Stasik 2017; Szołucha 2018b; Stasik 2019), often seeking to illuminate the scientific democratization of society and the production of expertise. Historical reconstructions of Europe’s “affair” with shale gas have been attempted from early on (Terral 2012). Economic analyses, such as Geoffron’s (2018), questioning the profitability of shale gas extraction within the 2015 French Ecological Transition Act legislation, have also been conducted.

Sociological research has mainly focused on the narratives produced by the press and other media outlets, as well as those formulated by political parties and think tanks (Jaspal & Nerlich 2013; Jaspal et al. 2014; Mercado et al. 2014; Cotton et al. 2014; Lis & Stankiewicz 2017; Metze 2017; Lis 2020). Other work has examined anti-shale and anti-fracking mobilizations (Steger and Milicevic 2014; Terral 2014; Weible et al. 2016; Szołucha 2021). Within this literature, Poland has attracted attention especially from political scientists, whereas studies on France span a broader range of disciplines. In both cases, historical reconstructions have often been intertwined with political analyses: Materka (2012a) was arguably the first to try to understand Polish events through an analysis of resource nationalism, while an early historical reconstruction of the French case, focusing on the rapidity of citizen mobilization, can be found in Terral (2012).

Cantoni et al. (2018a) have reconstructed and analyzed the evolution of shale-gas-based narratives in both France and Poland, revealing common promotional strategies—economic benefits, improved energy security—but also country-specific differences. Promoting gas as a “bridge-energy” from coal to renewables worked much better in coal-dependent Poland than in nuclear-dependent France, whereas maintaining that shale gas would allow for a revitalization of the national oil and gas industry worked better in France, which has a long-standing interest in this area. Since then, other studies focusing on Poland have examined various aspects related to the democratization of decision-making (Materka 2012b; Stankiewicz 2013; Stankiewicz et al. 2015; Szolucha 2018a) at the national and local levels, the negotiation of local governance (Lis and Stasik 2017), and the creation of energy citizenship in local communities mobilized against shale gas (Cantoni et al. 2018b). This study expands on the latter work but concentrates on the creation of technoscientific expertise.

Reflections on issues of technoscientific expertise are also present in other research on France: Chateauraynaud & Debaz (2013) have shown how social actors can enter an estimate-producing space monopolized by industrial actors and take advantage of it to make decisions they deem necessary for the exercise of their autonomy. Baudrin et al. (2014) have investigated how, after the passing of the 2011 Jacob Law, industry representatives have organized themselves to keep the controversy open by redefining the parameters of the debate. Molinatti and Simonneau’s (2015) study focuses on the discourses, practices, and representations of a group of French scientists who made public statements about shale gas during the controversy, and on how their interventions led them to reflect, both as individuals and as members of a community, on the ways in which they represent science in society. A corollary of their finding is that opposition to or support for a specific technology may depend on the broader ideological views of each practitioner. Chailleux’s (2016) study focuses on the production and reduction of uncertainty during the debate, while Keeler’s (2016) and Chailleux and Moyson’s (2016) examines shale gas politics and anti-shale gas movements in France. Like Baudrin et al. (2014), Chailleux & Moyson also emphasize the power of national technocratic elites in keeping the debate open in hopes of future political or economic change.^1^ Finally, Aczel et al. (2018) have explored the extent to which public participation in decision-making played a role in French fracking regulations.

These considerations, together with the attempts made by citizen committees and NGOs to enter decision-making spaces concerned with environmental and energy issues, have enabled the gradual structuring of what geographer Patrick Devine-Wright has called “energy citizenship”: that is, “a view of the public that emphasizes awareness of responsibility for climate change, equity and justice in relation to siting controversies as well as fuel poverty, and finally, the potential for (collective) energy actions […]” (Devine-Wright 2007: 68). An important part of the concept of energy citizenship is the bottom-up production of counter-expertise. Since in many environmental disputes the only factor influencing the outcome of a struggle is the ability to produce knowledge, shale gas development can serve as a particularly generative field of research.

One way to exert energy citizenship is through the production of scientific expertise by communities.^2^ Since the 1990s, such expertise has been one of the core objects of investigation for researchers in STS. In a context of post-normal science—that is, a science in which “facts are uncertain, values in dispute, stakes high and decisions urgent” (Funtowicz & Ravetz 1993: 744)—scholars such as Steve Epstein (1995), Sheila Jasanoff (1995), and Brian Wynne (1992, 1996) have argued that lay experts are capable of “extensive informal reflection upon their social relationships toward scientific experts, and on the epistemological status of their own ‘local’ knowledge in relation to ‘outside’ knowledge” (Wynne 1992: 281). By “lay experts,” researchers mean people who do not have a formal scientific background but whose situated experiences have helped them develop alternative forms of knowledge on particular topics.

However, this “non official” science, produced outside of mainstream institutions, should not be considered the exclusive domain of lay experts but as a field accessible to a broad set of actors, ranging from retired experts to consultants. This becomes especially clear in the case of France, where older citizen-activists, who had already taken part in earlier science-based controversies, such as those surrounding GMOs and nuclear power, reactivated their personal and community networks, which often included academic “maverick” scientists. These actors were instrumental in helping French protesters develop forms of energy citizenship. In both the French and Polish cases, the production of “unofficial” scientific expertise, or counter-expertise, played an important role. This kind of expertise was used to obstruct government-sanctioned exploration operations and contributed to the eventual decision of most companies to withdraw from potential extraction sites. The shared production (or co-production) of this knowledge occurred in the virtual and physical spaces which Callon et al. (2001) have termed “hybrid forums,” and which represent a new form of dialogic democracy.

Using two case studies revealing the co-production of knowledge in technoscientific mobilizations, I show how episodes of resistance to unwanted energy projects contributed to the creation of networks of competencies that could be mobilized by citizens in their attempts to oppose official narratives as well as knowledge produced in formal institutional settings. I show that the communities involved in shale gas controversies gradually acquired the ability to conceive of energy infrastructure as a sociotechnical system. In addition, the exchanges of information between activist groups and specialized NGOs, as well as among activist groups from different European locations, which was made possible by the use of information and communication technologies, allowed for the coming together of a genuinely transnational, science-based anti-fracking movement. Finally, I show how the characteristics of the two mobilizations reveal the French one to be national and science-focused while the Polish one can be described as local and focused on both science and law.

### Methodological Note

The primary information this article is based on was gathered during 40 semi-structured sociological interviews (20 per country) conducted between the fall of 2015 and the spring of 2016. Interviews lasted between two and three hours, with different types of stakeholders selected through a survey of press coverage followed by snowball sampling. The selected interviewees included industrial and institutional actors, activists, and members of environmental NGOs and think tanks. The two target areas were the department (*département*) of Ardèche in the Auvergne-Rhône-Alpes region in southeastern France and the administrative commune (*gmina*) of Grabowiec, part of the county (*powiat*) of Zamość, in the Lublin region, in southeastern Poland. However, interviews were also conducted with stakeholders in areas not directly involved in the initiatives under discussion, including the Parisian metropolitan area, Warsaw, and Cracow.

Aside from their sociological function, the interviews also had a historical one, allowing the interviewees to present their own narrative reconstructions of relevant events. As such, they made it possible to recover information that had escaped prior press coverage. Interviews focused on the production and use of technoscientific expertise and counter-expertise, as well as the exchanges of information that made it possible. The interviews were qualitatively analyzed using Atlas.ti® software.

## The Production of Shale Gas and Fracking Counter-Expertise in France and Poland

Since research on both countries has already reconstructed their respective histories with shale gas extraction, I will provide an outline of the main events in Table 1 (France) and Table 2 (Poland), drawing on published sources as well as my own research, and refer to them where necessary.

**Table 1:** Summary of main events in the French shale gas controversy. (Adapted from Chailleux & Moyson 2016)

Date	Event
2008	Issuance of several exploration licenses for shale oil, involving fracking
March 2010	Issuance of three exploration licenses for shale gas, involving fracking
October 2010	News of the three shale gas permits made public by a *Charlie Hebdo *journalist
From December 2010	Distribution of *Gasland*, a critical documentary on shale hydrocarbons and fracking
	Growing social and political mobilization against fracking
January-March 2011	Departmental elections: anti-shale mayors win in many French towns
February 2011	First national anti-shale rally at Villeneuve-de-Berg (Ardèche county): 15,000 participants
March 2011	Socialist Party (center-left, minority party) introduces a bill proposing to ban fracking
	UMP (center-right, majority party) introduces a similar bill (“Jacob Bill” proposal)
May 2011	Publication of a parliamentary report on the Jacob Bill (Havard-Chanteguet report). Favorable to a ban
June 2011	Publication of the report of a Parliamentary Committee (Gonnot-Martin report). Key conclusions: Lack of knowledge on shale resources; inadequacy of subsurface mineral rights regarding companies’ needs and citizens’ rights
July 2011	Passing of the Jacob Bill, resulting in ban on fracking (but not on shale oil and gas exploration)
August 2011	Demonstration at Lézan (Gard county): 15,000 participants
October 2011	Cancellation of the three controversial permits assigned in March 2010 (followed by litigation between State and companies over the next months)
	Demonstration at Barjac (Ardèche), gathering 7,000 people
February 2012	Publication of the report of the administrative CGIET-CGEDD Committee. Key conclusions: Lack of data on shale resources; inadequacy of current mining legislation; “clean” exploitation of shale resources could be attempted
April–June 2012	Presidential and general elections: Socialist Party’s candidate (F. Hollande) wins presidency; Socialist Party wins parliamentary majority
July 2013	Hollande declares no shale gas exploration to occur in France under his presidency
October 2013	Constitutional Council confirms validity of fracking ban
November 2013	Publication of the report of the parliamentary OPECST Committee (Lenoir-Bataille report). Favorable to fracking in developed countries with high environmental requirements. Suggests: Encouraging experimentation; exploration and assessment of French resources
August 2015	Passing of Law n. 2015-992 on energy transition
January 2016	Total recovers its cancelled permit in southeastern France but declares not to take active steps in the near future
May 2016	Environment and Energy Minister (S. Royal) declares intention to prohibit imports of shale gas to France from the US
December 2017	Passing of Law n. 2017-1839: end to hydrocarbon exploration in France by 2040; prohibition of exploration for shale hydrocarbons

**Table 2:** Summary of main events in the Polish shale gas debacle

Date	Event
2007	First issuance of exploration permits in Poland Grabowiec permit issued to Lublin Energy Resources (later, acquired by Chevron)
September 2010	First seismic tests performed by Geofyzika Toruń
August 2011	Lane Energy fracked Poland’s first well (Łebień LE 2H in Pomerania)
December 2011	Arrival of Chevron representatives in Grabowiec
January 2012	Meeting between Grabowiec residents and Chevron representatives
March 2012	Publication of the Polish Geological Institute’s report on national shale resources. *Key finding:* Higher probability range of recoverable shale gas resources: 346–768 Bcm
	Chevron gives up first attempt to begin activities because of EU’s bird mating regulations
June 2012	Chevron’s authorization for test drilling found incorrect: it only allowed for seismic tests Exxon Mobil cancels Polish shale gas projects
October 2012	Government proposes new taxation schemes and geological regulations: standstill
June 2013	Plots leased by Chevron occupied “Occupy Chevron” blog opened
January 2014	ENI withdraws from Poland
March 2014	Government approves new legislation exempting gas companies from paying royalties until 2020 for the extraction of shale gas
April 2014	Total withdraws from Poland
June 2014	Significant drop in oil price, lasting until January 2015 (from $105 to $54 /barrel)
January 2015	Chevron withdraws from Poland
June 2015	ConocoPhillips withdraws from Poland

### Ardèche: A Network of Collectives in a Professionally Diversified Area

**Fig. 1:**
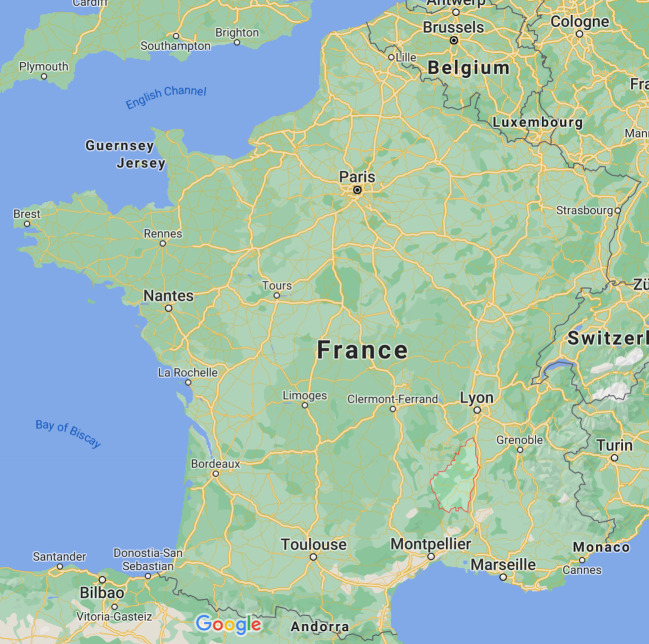
Map of Ardèche within France. (Image source: Google Maps, 2021)

No French source, either among activists or among the coalition of pro-shale actors, failed to mention *Gasland* as being among the main reasons for the local population’s early skepticism of shale gas extraction. Indeed, the documentary proved to be a formidable tool of persuasion. That it contained many inaccuracies was recognized even by activists; that it was a valid narrative tool was acknowledged even by industrial and institutional actors. Scenes from the film were shown in virtually all of the initial meetings of the anti-shale collectives that had begun to form throughout France in late 2010, but particularly in the country’s southeast. The Collective 07 “Stop shale gas” (*Stop au gaz de schiste*) in the Ardèche department was among the most active in this regard (a map of Ardèche is provided in Fig. 1). Jacqueline Balvet, a leading member of the collective and of ATTAC France, claimed: “We made excerpts because he [Josh Fox] allowed us to do what we wanted with it. […] We had a ten-minute and a twenty-minute excerpt, and it’s true that, since we showed those images everywhere … We went around a bit in all the towns. It made an impression on people.”^3^

However, it was not just *Gasland* that triggered the sudden politicization of local residents. Ardèche county was already a markedly politicized area by the time the shale gas debate started. In the 1970s, the neighboring Larzac area witnessed successful struggles against the extension of a French military base. In these struggles, José Bové, who later became one of the most important figures in the French ecological movement, stood out. One of the three permits for shale gas exploration (the Villeneuve permit), applied for and obtained by the American company Schuepbach, included Larzac. Together with the lack of transparency in the communication of the allocation of permits (news of which appeared in the press seven months after their assignment), the prospect of fracking in southeastern France immediately reactivated existing networks of activists in the region.^4^ Indeed, the early core of the French movement was formed by activists who had participated in similar protests in the past. The movement could count on lessons learned from past issues (GMOs, nuclear energy, the military base project) and use them to rapidly formulate a set of arguments in their support. These mainly left-leaning activists were soon joined by local authorities, including from the governmental center-right party, who took a firm stance with respect to fracking activities, organizing local discussions, sending petitions to the national government, and even allowing protesters to hang anti-shale banners from local town halls. The mobilization came together quickly and received active or passive support from significant parts of the local population.

A second aspect of the mobilization was the ability of actors to depict specific problems as part of larger issues. The protest, born of local anxiety and discursively centered on local territories, soon began to include more general demands, summarized in the slogan: *Ni ici, ni ailleurs. Ni aujourd’hui, ni demain* (“Neither here nor elsewhere, neither today nor tomorrow”). Collaboration and discussion among the participants also allowed for the incorporation of more radical issues, such as alternative concepts of growth that focus on psychosocial rather than economic metrics. As noted by Chailleux and Moyson (2016: 124): “Members of this anti-fracturing coalition succeeded in developing a base of shared beliefs related to environment, public health, and participatory decision-making.”

The first local anti-shale demonstration was organized by a small citizen committee of only eight people. It took place on December 20, 2010, in Saint-Jean-du-Bruel (Aveyron county), in the presence of 300 persons, including José Bové and Fabrice Nicolino, the *Charlie Hebdo* journalist who had revealed the existence of the three shale gas permits. The first online petition against shale gas was launched in the early weeks of 2011 and reached 50,000 signatures within a month (Terral 2012: 188). In January 2011, the first anti-shale committees were created in Ardèche as well as the neighboring county of Drôme, bringing together participants as different as environmentalists and sports hunters.^5^ The same month, the initiative began to transcend its local and regional roots when the first national mobilization, bringing together 15,000 people, took place in Villeneuve-de-Berg. A national coordination was then created, which gathered 180 collectives under its umbrella within a few months (ibid., 189), and which enabled the originally local movement to become fully national. The coordination acted on several levels, from the legal (aimed at the withdrawal of permits) to the technoscientific. Between January and March 2011, departmental elections were held in most French counties. In counties where shale gas was an issue, opposition to fracking became a basis for gaining legitimacy and was embraced by many successful candidates. Fearing the loss of power at the national level, and with presidential elections coming up, the ruling center-right party implemented a ban on fracking in July (known as the “Jacob Bill,” from its proponent’s name) and withdrew the three assigned permits later that fall. This marked a success for the anti-fracking movement. Total and Schuepbach appealed, and in January 2016, Total, which unlike Schuepbach had declared that it would not use fracking for exploration, regained its permit.^6^ However, the company decided not to proceed with exploration, justifying its decision with reference to low prices for oil and gas.

According to the French sociologists Francis Chateauraynaud and Josquin Debaz (2013), the intensity of the mobilization in France can be explained by the coming together of several factors: risks of pollution in areas perceived as pristine, a gap between recent environmental legislation and old mining legislation, and citizen interest in environmental issues which had been strengthened by a series of political meetings in 2007 (*Grenelle de l’environnement*).^7^ With respect to the first factor, most French shale gas projects were indeed located in agricultural areas. Additionally, shale gas extraction’s significant water needs, and reports of water mismanagement by US companies, did little to reassure locals about the safety of fracking for aquifers. Claims by French companies about the competence of French oil workers and strict European environmental regulations did not alleviate these concerns either. Interestingly, within the French oil and gas sector, blame for the French public’s reluctance to embrace shale gas drilling was put as much on the French citizens’ supposed ignorance of science (echoing the widespread but highly criticized information deficit model) as on the incompetence of US companies, which they thought had given fracking a bad name.^8^ As a consequence, the production of unconventional gas was quickly framed as incompatible with local narratives about water use and food production.

Even in the early stages of mobilization, activists were aware of the need to oppose shale advocates not only with legal but also with scientific tools. As argued by Theodore Porter (1995) and Steffen Mau (2019), since World War II, reliance on quantitative methods has gradually replaced reliance on qualitative ones, making the former the yardstick for credibility. As a consequence, opposing industrial developments solely on ideological, aesthetic, political, or social grounds is no longer deemed an effective protest strategy —particularly since government and industry representatives can rely on a powerful apparatus for producing data, statistics, and models. This is especially true for the French case, where, as argued by Baudrin et al. (2014: 475), following the passage of the Jacob Bill into a law, institutions sought to limit the relevance of experts to purely scientific arguments. Activists countered this attempt to limit their participation in the debate by producing their own expertise. In this sense, science was both an offensive and defensive tool.

The socio-economic structure of the Ardèche also played an important role in these developments. It is a place known for attracting retired professionals from other regions of France, who oftentimes move there in search of tranquility and contact with nature. Several of the activists of Collective 07, for example, were not originally from the Ardèche, having developed their professional skills elsewhere and in sectors other than those prevalent in the region (agriculture and tourism). This enabled the formation of a critical mass of heterogeneous professional skills, the coming together of which was essential for the formulation of scientific counter-narratives, especially in the field of hydrogeology, an area often neglected by the oil and gas industry (Chailleux 2016: 530). For example, former teachers, civil servants, oil technicians, and even an oil geologist who had worked in the industry, were all part of the effort. Within the collective, a scientific committee was formed which allowed other activists to educate themselves with the help of people with firsthand experience.^9^ Both in terms of perception and practice, this enabled participants to gain a substantive understanding of the issues involved and to counter accusations of amateurism coming from “official” experts.

In addition to the expertise of the collective’s own members, the mobilization in Ardèche could also count on allies in other locations, including at grassroots organizations, NGOs, and academic institutions. At the international level, anti-fracking activists, strengthened by the support of José Bové, who was a member of the European Parliament at the time, were able to not only organize meetings and exchange information with Polish, Bulgarian, German and British groups (see below), but also to connect with Canadian activists as well as the association No Fracking France (Terral 2012: 193).^10^ Chailleux (2015: 56) argues that the work of activists from Quebec was reused in France and that the French Jacob Law in turn affected shale extraction in the Canadian province.

The anti-shale movement also received institutional support, and it is at this point that counter-experts came into play. Chailleux (2016: 532-3) mentions a 2011 report by geologists and hydrogeologists from the University of Montpellier, which cast doubt on the profitability of gas extraction in the southeastern France by pointing out that the karst nature of the local geology would make it difficult to properly cement wells. One of the group’s hydrogeologists, Séverin Pistre, would later become an important participant in this debate by challenging the geological arguments used by the Ministry of Ecology’s Bureau of Hydrocarbon Exploration and Production. Indeed, as Chailleux and Moyson (2016: 125) note, at the national institutional level, “civil servants were divided between an anti-fracturing and a pro-exploration position. This position depended on, among other things, their institutional affiliation.” State geologists and engineers typically tended to see fracking in terms similar to those of engineers employed by mining companies: as a safe and well-established practice. By contrast, hydrogeologists were significantly more cautious, as were environmental scientists. The testimony I collected from industrial actors and from national geological or petroleum institutions often includes the argument that researchers ought to be able to do their jobs: that is, they should be allowed to engage in subsurface exploration to gather more information. However, opponents of shale gas extraction feared that exploration would inevitably give way to exploitation should the tests indicated the presence of commercial-value gas. Activists seemed to lack confidence not just in the willingness of industrial actors to follow the law but also in the ability of government authorities to effectively oversee them.

The formation of counter-expertise within Collective 07 occurred in concurrence with the action of the association No Fracking France, but often without the existence of a shared strategy. Indeed, as noted by Chailleux (2015: 275), according to one of its members, No Fracking France was created in opposition to the strategies of the collectives. Whereas the collectives tended to focus on demonstrations and civil disobedience, No Fracking France promoted a vision of opposition that relied on science and expertise. In addition, members of No Fracking France opposed the left-wing political orientation of the collectives, which they thought hindered their ability to claim objectivity in their arguments.^11^ With these intentions in mind, No Fracking France organized a series of conferences (mainly in Paris) on shale gas and fracking in the fall of 2011, aimed at elected representatives and attended by hydrogeologists (Pistre) and toxicologists (Picot) as well as lawyers, physicians, political scientists, and economists. In November 2011, the Scientific Interest Group Envirhônalp—a network of 80 laboratories in the Rhône-Alpes region carrying out research on the environment—organized a meeting on shale gas together with Grenoble’s Institute of Earth Sciences, inviting a similar roster of experts. This meeting was attended by No Fracking France representatives as well as by some representatives of the southeastern collectives.^12^

Beyond the conferences in Paris organized by large ecological associations, collectives also created their own repositories of knowledge through online research. According to Jacqueline Balvet, executive member of the Association for the Taxation of Financial Transactions and Citizen’s Action (ATTAC) France, the formation of expertise took place in part through web research, the results of which were then discussed during meetings of the collectives. The counter-expertise created by the members of the collectives, especially those familiar with scientific issues, translated the macro-level discussions of the meetings in Paris to micro-level conversations in particular local places.^13^

In September 2012, No Fracking France organized a fact-finding visit to Pennsylvania and Quebec, to allow activists as well as elected representatives of the General Council of the department of Lot-et-Garonne (southwest France) to observe the effects of shale gas extraction firsthand (Chailleux 2015: 276). Chailleux (2015: 170) maintains that the Quebec visit was made possible by former collaborations between the two activist communities, which had also included the exchange of scientific information as well as personnel. For example, Pierre Batellier, professor of economics at HEC Montreal and a French national, as well as a member of the Saint-Marc-sur-Richelieu (Quebec) collective’s scientific committee, took part in the Villeneuve-de-Berg demonstration in France. Later, Lucie Sauvé, who belonged to the same committee, traveled to meet the members of the French collectives. The co-production of such knowledge led Robert Pilli, the coordinator of the Rhône-Alps Collective “Stop shale gas,” to emphasize the idea that activists had a right to speak and be heard: “So, if we speak and if we continued to speak it is because we had tangible elements, […] and we have the right to demonstrate that indeed the oil companies, the industrialists on this kind of situation lie all day long.”^14^

Generally, however, both industry and government representatives dismissed any kind of knowledge produced outside established institutional settings. This created a sense of frustration within the collectives, a situation that is reflected in the words of Alain Souléliac, a former entrepreneur and Collective 07 activist:

It’s complicated to extract in Europe. Everybody knows that! […]. In the collective, we have people who are hydrogeologists, we have people who have worked in oil, that’s a little bit the citizen force. We are at the bottom of the Ardèche but there are not only stupid people here! There are not only fools, uncultured, ignorant people with hooves and straw in their hair! We all have … there is a cultural and intellectual level within the Collective, which is very, very impressive!^15^

Picot’s role in the production of counter-expertise was stressed by Pilli, who credited the toxicologist with the idea of organizing a fact-finding mission to North America to gain more information about the chemicals contained in fracking fluid. Upon his return, Picot argued that while information about the chemicals used in fracking fluid was available, there remained uncertainty about how they would interact with forms of life and elements already present in the subsoil. Such interactions, he argued, could generate new chemical substances with unknown consequences for both the environment and human health.

Another scientist who contributed to the production of scientific expertise in southeastern France was Jacques Cambon. A hydrogeologist by training, and formerly employed by the research department of Suez-Lyonnaise des Eaux, a French company specializing in water and wastewater services, he eventually became a full-time member of ATTAC France.^16^ After watching *Gasland*, he participated in a meeting on shale gas in the Gard department, close to Ardèche, in the summer of 2011. Gard was included in the Montélimar permit, one of the three permits issued in 2010. At the meeting, Cambon met activists from Gard, who showed him maps of the permits which indicated that they extended to the departments of Savoy, Upper Savoy (where he lived), and Ain. He claims that he immediately understood what was at stake: “For me water is, it has been my whole professional life and the risks of pollution were the first thing I saw: the risks of water pollution, so we had to fight to protect our own resources.”^17^ In response, he decided to set up a collective in collaboration with members of the agricultural trade union (*Confédération Paysanne*), the Rhône-Alpes Federation for Nature Protection (*Fédération Rhône-Alpes de Protection de la Nature*), and Friends of the Earth’s French chapter (*Les Amis de la Terre*). Similar to other collectives, its aim was to inform both the public in general and elected representatives in particular about the risks associated with shale gas extraction.

Both Cambon and Pilli mentioned their reliance on confidential documents produced by the Bureau of Geological and Mining Resources, which they had managed to obtain, and which contained information about the dangers related to the accidental upswelling of gas in the subsoil due to the presence of buildings and drainage systems.^18^ The importance of protecting water resources in the Ardèche, a region affected by chronic drought, was an important theme in the argumentation of the activists.

The anti-shale movement also received help from the federation of ecological associations, France Nature Environnement (FNE), although it did so mainly on a legal rather than a scientific basis. FNE helped disseminate information by organizing meetings on issues such as shale gas, the mining code, and political lobbying. It contributed to a report (the “Tuot Report”), commissioned by Prime Minister Jean-Marc Ayrault in 2013, on the reform of subsurface mineral rights, which in turn led to the presentation of a bill aimed at creating a High Council of Mines in 2015.^19^

What this history shows is that the process of counter-expertise co-production—especially through the publication of data on the karst nature of the subsoil in the Southeast and the distribution of analyses casting doubt on the profitability of fracking in this region—permitted activists to counteract pressure from shale advocates and prevent closure of the debate. Exchanges between the Ardèche collectives and other actors forced the former to discuss, debate, and compromise with the latter. This can be understood as an exercise in deliberative democracy and energy citizenship within a hybrid forum. Most of the time, these forums were physical spaces, such as conference halls and NGO headquarters in Paris or rented venues belonging to municipalities in the Ardèche. But virtual spaces on the internet were also used by the activists. Nationwide discussions were as frequent as local debates, and international links to North American and other European activists not only helped amplify the Ardèche protest but also permitted the acquisition of second-hand experience from places that had already experienced fracking. The dominant element in the co-production of knowledge was technoscientific, whereas legal issues were addressed by a smaller community of professionals, especially after passage of the Jacob Law. This makes the French case markedly different from the Polish one.

### Żurawlów: A Virtually Connected Island Bridging the Local and the Transnational

**Fig. 2:**
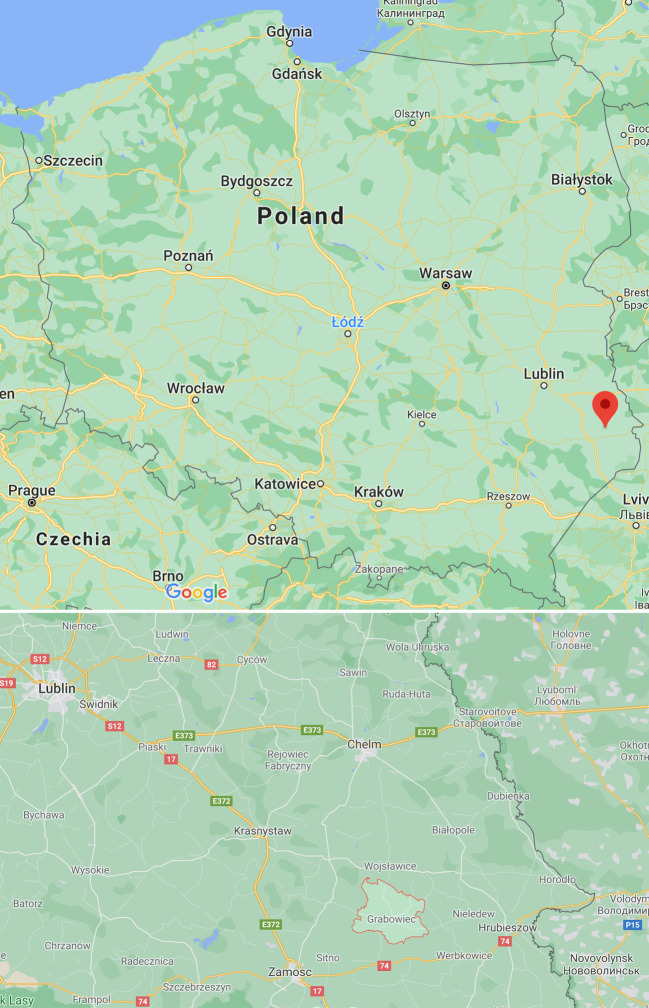
Map of the Grabowiec municipality within Poland. (Image source: Google Maps, 2021)

The political mobilization of the small village of Żurawlów, located in the municipality of Grabowiec, in the county of Zamość, in southeastern Poland (Fig. 2), was an unusual phenomenon for that country. While protest movements also formed in Poland’s other region affected by shale gas activities, Pomerania, the events in Żurawlów (spreading to the neighboring villages located in the same region) received significantly more media attention. By delaying corporate exploration activities, residents achieved what they had mobilized for, and Chevron eventually left not just the village but also the country. Whether the Żurawlów initative was the main reason for the company’s retreat is an open question: the drop in the oil price beginning in mid-2014, the economically low estimates of the region’s extractive potential, and the constantly changing regulatory regime likely also played a part in Chevron’s decision. What the protest did do, however, was produce a critical mass of anti-shale counter-expertise, help strengthen activist connections at the national and international levels, and play a crucial role in the creation of local energy citizenship. The main events in Poland’s shale gas story are summarized in Table 2.

The permit covering Żurawlów was assigned to Lublin Energy Resources (later acquired by Chevron) in 2007. Chevron’s geophysics subcontractor, Geofizyka Toruń, performed seismic tests in September 2010, and in December 2011, representatives of the US company arrived in Grabowiec to consult with mayors and acquire local support. The company also organized an informational meeting in the village of Horodysko, about 20 km from Żurawlów, where shale gas drilling already took place. The trip was framed as an educational activity (Cantoni et al. 2018b).

At the same time, residents of Żurawlów, including Barbara Siegieńczuk, the owner of an advertising agency in the county’s capital, Zamość, and a participant in the Horodysko visit, started to gather information about the shale gas exploration and production sector as well as Chevron’s record. She retrieved news of the Lago Agrio oil spill, which had occurred in Ecuador in the 1990s, and which had led the local population to sue the American corporation in 2001. Chevron had been accused of dumping approximately 80,000 tons of oil and toxic waste in the Lago Agrio region between 1964 and 1990, which had led to increased rates of cancer, miscarriages, and other health problems.^20^ Unsurprisingly, information of this kind greatly contributed to the anxiety Żurawlowians felt.

A meeting between representatives of Chevron and local residents in January 2012 only increased the distrust the latter were feeling. The Chevron representative’s responses to residents’ questions about the technical aspects of the fracking process were seen as patronizing and condescending. The outcome of the meeting was opposite to what the company had hoped for. As Siegieńczuk recalls:

I knew from the previous meeting with Chevron representatives that they were assuring citizens that this water which is used in drilling […] consists only of water, sand, citric acid, and washing liquid. So, I asked the question: “What is in this liquid?” After hearing this answer, I said to them: “If you’re going to talk with us on that level, I take all the citizens and we just go back.”^21^

It was not just residents of Żurawlów who attended the meeting. Despite Chevron’s plans to make it a face-to-face meeting behind closed doors, representatives of environmental NGOs and journalists were also present for the occasion. The media had been summoned by Teresa Adamska, the co-director of the Center for Sustainable Development (*Centrum Zrównoważonego Rozwoju*, CZR). Its presence, together with that of the activists, annoyed Chevron representatives so much that they decided to leave (Stasik 2018). In this case, it is clear how communication between local and national organizations empowered the involved citizens. Without the presence of the NGOs and the media, the clash between the residents and the company might have gone unreported, foreclosing the opportunity for the protest to grow and expand. The meeting convinced locals that they needed to monitor both the company as well as local authorities. The trans-scalar nature of the mobilization became visible once more when residents turned to environmental NGOs in large Polish cities, as well as to the Polish Green Party, for help (Cantoni et al. 2018b). These organizations provided activists with further resources that could help them stop, or at least slow down, the company’s operations in the area.

The territory southwest of Żurawlów is home to several forests (the National Park of Roztocze, the Puszcza Solska forest, the Szczebrzeszyn Landscape Park) which are protected under the European Union’s “Natura 2000” regulations. Natura 2000 required preliminary environmental impact assessments (EIA) for these sites, to ensure that economic activities would not threaten their ecological health (Fleming 2017: 91). Aside from CZR, two other NGOs provided further legal help and environmental information: INSPRO and Eko-Unia. The urgency of collecting data before Chevron could begin operations led to a frenzy of exchanges between the NGOs and the local community. As CZR’s Adamska recalls: “All the time my phone was ringing. We had no private life and we lived with the life of Żurawlów.”^22^

To slow Chevron down, Eko-Unia advised the residents of Żurawlów to make use of a law prohibiting the start of economic activities during the breeding seasons of specific wild birds. The EU’s “Birds Directive” (Council Directive 2009/147/WE), meant to protect animal habitats, restricts industrial development between March and July. This strategy proved effective, stopping Chevron’s activities for several months beginning in March 2012. Later, the London-based NGO ClientEarth used its Warsaw branch to produce the *Black Paper* report, which provided the anti-shale activists with additional support. The report criticized Poland’s implementation of the European Union’s climate and energy regulations, especially with respect to a bill that would prevent new NGOs from participating in EIA procedures (Stoczkiewicz 2013). The bill’s implicit goal was to exclude anti-fracking organizations that had been founded after the issuance of the permits.^23^

Żurawlów gained additional media attention when Lech Kowalski, an American filmmaker of Polish descent, visited the area in April 2013. Kowalski took several residents of Żurawlów to Cracow to watch his film on fracking, *Drill, Baby Drill*, which was screened at a documentary film festival. When visiting Żurawlów at the beginning of June, he became aware of the tensions between local activists and Chevron, and soon after took on the role as the protest’s international spokesperson. He urged the activists to set up an internet connection in the village, so that news from there could reach other activist groups in Europe and beyond. Kowalski’s partner, Odile Allard, a French national, set up a trilingual website in Polish, English, and French for what would become known as the “Occupy Chevron” movement. Once more, interaction between local and international actors helped bring a level of attention to the mobilization that would not have been possible otherwise. The website, which became an important venue for exchange and debate, was soon taken over by Polish volunteers from an urban squat (Sufin-Jacquemart 2015).

Chevron made a comeback in June 2013, but when company workers returned and started erecting a fence around the company’s plot of land, the population mobilized physically and legally. They used tractors to block the road to the area and checked the details of Chevron’s permit. When they discovered that the permit was limited to seismic tests, which had already been conducted, and that the authorization to drill had been cancelled a year earlier, they argued that there was no necessity for the fence.^24^ Demanding that Chevron stop the project, protesters began to occupy the company’s plot. Soon after, activists set up a website, a Facebook page, and a YouTube channel to document and give visibility to what they were doing. Activists from around Poland and nearby countries also came to the aid of the protesters. Aside from enabling the co-production of scientific knowledge, the mobilization also, as in the French case, brought together actors that would have not come into contact otherwise.

As the movement grew in size, so did its repertoire of arguments. Following suggestions by a Czech activist, a retired Polish hydrogeologist, Andrzej Szczepański, encouraged citizens to consult maps of the country’s water systems showing that Żurawlów was located over one of three largest underground reservoirs in Poland. He demonstrated that four of Chevron’s permits lay entirely within the area of the largest groundwater reservoir in the region of Lubelszczyzna. EU directives specified that groundwater reservoirs had to be protected. But Poland lagged behind in implementing these regulations, and because no protected areas had been established so far, the permits had technically been issued in accordance with the law (Sufin-Jacquemart 2015: 43).

This, of course, did not decrease the potential danger. Mismanagement of fracking operations, or the simple reactivation of a naturally active fault in the Lubelszczyzna region, would be able to cause pollution affecting the whole reservoir.^25^

In the French case, the importance of water was linked to the issue of drought in Ardèche, and preserving soils was seen as crucial for a region heavily dependent on both agriculture and tourism. In the Polish case, by contrast, the nexus between water and soil lay in the soil quality of the Lublin region, which is among the highest in Poland, and which contributes in crucial ways to its economy (European Commission 2022). Therefore, the whole regional economy would suffer from a reduction of the area’s environmental health. The land-water-food nexus was a link the residents could understand immediately and intuitively.

Polish proponents of shale gas extraction, like their French counterparts, accused local citizens of scientific ignorance and tried to establish a monopoly over the production and distribution of geological knowledge. Their perspective was widely represented in the media. The creation of an epistemic monopoly was also, in Siegieńczuk’s view, the main goal of the meetings that the local government organized with shale gas opponents. One of these, which was part of the “Let’s talk about shale” campaign co-financed by the European Union, took place in Lublin in the fall of 2013.^26^ However, Siegieńczuk explains that “they didn’t succeed, because we were more competent than they thought. We were always taking part in discussions, we were interviewing scientists, hydrogeologists, and I’m very familiar with the subject.”^27^ In the event, Chevron’s lawyers, as well as government representatives trying to speed up legal procedures, frequently found themselves having to counter expertise produced at the community level.

The people of Żurawlów also developed a transnational network connecting them with individuals and organizations from other countries and continents. They welcomed activists, politicians, and journalists, and travelled to conferences, seminars, debates, and hearings. A delegation from Żurawlów attended a major conference on shale gas in the European Parliament, where they met *Gasland*’s director, Fox. They also visited the Polish Parliament’s lower house to participate in consultations with the Committee on Agriculture and Environment. A Żurawlów village representative accompanied Kowalski to Balcombe in England, where Cuadrilla had planned to start drilling. A few days after watching Kowalski’s film and exchanging information with the Żurawlów delegation, Balcombe activists started their own demonstration in July 2013 (Sufin-Jacquemart 2015).

For his part, Kowalski used his association with Bové to draw attention to the cause of Żurawlów’s residents. Bové had already participated in a conference on the damages caused by shale gas extraction in Wrocław.^28^ Adamska, who was also present at the meeting, had recorded it and created a presentation in Polish that was then posted on YouTube. “Thanks to our presentation, most people in Poland knew what the threats of shale gas [are] and what it is. The video reached over 500,000 views within two months,” she recalls.^29^ So, when the residents showed Kowalski the documents proving that company did not have a valid drilling license, Kowalski informed Bové, who later visited Żurawlów, together with activist leaders from England, Germany, and the Czech Republic. According to Sufin-Jacquemart (2015), they also sought the support of local politicians and Members of Parliament—unsuccessfully, as it turns out, since all Polish political parties supported shale gas exploration. However, the demonstrators did receive support from the Greens/European Free Alliance (EFA) group in the European Parliament. The Alliance’s president, Rebecca Harms, met the activists at a conference in Warsaw. The Polish Greens also visited Żurawlów regularly. Lacking seats in parliament, however, they did not attract media attention.

As the Żurawlów mobilization gained visibility, and as anti-shale protests multiplied the world over (Steger and Milicevic 2014; Terral 2014), a dramatic fall in the price of oil and oil-indexed natural gas upset global markets, starting in mid-2014. In combination with the technical problems other companies had encountered as a result of the country’s specific geology (Cantoni 2018), this sudden decrease in profitability further added to the regulatory instability of Poland’s geological and mining sector. Taken together, these developments spelled doom for the fracking industry in Poland and elsewhere in Europe. In July 2014, Chevron made the decision to leave Żurawlów, and its lease on the plot was cancelled. However, during the dispute, the company had numerous taken residents of the village to court, bringing eight cases against 34 farmers (The charges were dropped after Chevron departed.). In the final analysis, the tactics deployed by the activists helped delay the company’s operations long enough for external events to make the extraction of shale gas unprofitable.

## Conclusions

This article has focused on the production of counter-expertise, particularly technoscientific counter-expertise. As I have shown, this was not the only kind of expertise mobilized by opponents of shale gas extraction. Depending on the setting in which they operated, legal and economic expertise were also used. However, in the modes of opposition conceived by activists in the two case studies presented here, there was, from the earliest stages of their protests, a clear understanding of the importance of scientific evidence. Activists therefore proceeded, independently at first and with the help of associations and NGOs later, to acquire data and information that would allow them to cast their opposition in scientific terms. This was done with the explicit intention of counteracting attempts by technocratic elites and corporate actors to monopolize techno-scientific discourse. Using science to problematize fracking, French activists were able to leave a “window of uncertainty” open and to keep the debate alive even after the Jacob Law passed.

In the Polish case, by contrast, where the general mood favored the exploration and extraction of shale gas, any windows of uncertainty at the national level were virtually closed from the beginning. Activists therefore operated at the local scale, the only one that allowed for targeted action. At the same time, however, they made use of trans-scalar collaborations. While the extent to which the French mobilization expanded territorially can be understood as a result of previously existing networks of lay expertise, which were themselves the outcome of earlier controversies over different issues, the dynamics of the Polish mobilization are different. After all, the area in which Chevron had planned to conduct its activities had not previously been a site of a large protests. In the case of Żurawlów, arguably, it was the international resonance and the support that the protesters gained early on that made it possible for them to create a network able to provide them with the scientific information needed to oppose the “official” narrative.

While in the French case, the principal sites of knowledge production and information exchange were physical, in the Żurawlów case, they were as much virtual as physical. In addition, unlike in France, where scientific evidence dominated the arguments of the movement, the Żurawlów protestors relied on a mixed approach. The help they received from two NGOs with legal expertise formed an important element of their movement. Yet this legal expertise was based on environmental knowledge, which means that science, yet again, played a crucial role in their work.

Another difference between the two cases has to do with their respective geographical scales. While the French movement expanded rapidly from the local to the national level, in part because of the presence of multiple sites of anti-shale action in the country, the Polish movement remained largely local, operating at the national scale only when it came to the gathering of scientific and legal evidence. While the residents of Ardèche could count on a swift reactivation of previous knowledge networks, those in Żurawlów needed to build their network from scratch.

The development of counter-expertise was the result of collective efforts on several levels. Collaborations between local, national, and international actors were frequent and fruitful. These collaborations increased these actors’ identification not only with their regional and national communities but also with a broader European collective, empowering them in their particular forms of energy citizenship. In most cases, protests also increased the scientific and political awareness of participants, allowing them to become more active decision-makers in the places they live. The co-production of expertise involved various types of people, including active and retired scientists, legal advisors, bloggers, filmmakers, and young activists from urban contexts. This needs to be emphasized: aside from Wynnian lay experts and traditional institutional experts, a plethora of actors, with different degrees of scientific knowledge, participated in the controversy, sometimes using connections developed in their previous jobs to access confidential information.

Particularly important for the exchange of information across multiple geographic levels was the ability to draw on information from the internet. This medium, often viewed with condescension by official experts, enabled protestors to access a plethora of information, ranging from blogs to scientific research. Additionally, the internet also played an important role in connecting and mobilizing groups across space. Virtual communication was sometimes bilateral, as when collective videocalls took place. More often, however, it was one-directional and consisted of the simple retrieval of information from web pages. Use of internet was particularly significant in the Polish case, where, in the absence of a mobilization at the national level, the local and international dimensions needed to be bridged.

Transnational collaborations were also important for the formation of European energy citizenship, since they enabled activist groups to forge links, compare strategies, and disseminate information. In addition, the shale gas protests made citizens more aware of other hydrocarbons in their countries (for example, oil shales in the Parisian Basin or coalbed methane in France’s North and Lorraine regions), as well as the need to pay attention to them and to monitor the activities of extractive industries. The protests also created links—however tenuous and short-lived—between groups of stakeholders with very different goals, potentially leading to a widening of the political and social horizons of the actors involved.

Ultimately, the protests also led participants to ask broader questions about a number of issues, ranging from energy efficiency and consumerism to alternatives to economic growth and the place of humans in the ecosystem. In the words of Collective 07 member and ATTAC France executive Jacqueline Balvet:

What I find very, very interesting in this story of the fight against shale gas is that there was an absolutely extraordinary popular education, that is to say that we started from […] “I don’t want it in my garden”, and now there you have it, all the people who were in Barjac [where a mobilization was organized in February 2016][…] understood all the misdeeds of this industry, but not only of the shale gas industry, also of the fossil industry in general […]. The goal is resistance on these environmental issues: control of the environment, financialization of nature, etc. So here it is, things are progressing, and I believe that in five years we have made a rather extraordinary journey.^30^

## Footnotes

1 This was merely a temporary opening, however, since the newly elected French parliament adopted Law n. 2017-1839 in late 2017, which ends research for hydrocarbon extraction on French territory by 2040.

2 It is worth noting here that the concept of energy citizenship tends to focus on individual or community behavioral changes (Wahlund & Palm 2022), letting systemic changes on a macro scale remain in the background since these cannot be dealt with in individualistic terms but must be addressed as part of a broader discussion on energy ethics and macroeconomic models of production and consumption.

3 Jacqueline Balvet, executive member of ATTAC France, and activist of Ardèche’s anti-shale Collectif 07. Interview with the author, 04 Juy 2016, Aubenas. An interesting aspect of the distribution of *Gasland* in France concerns the way in which the film reached the anti-shale collectives of the Parisian Basin. According to Marie-Paule Duflot, a member of the Collective 77 Nature and Environment (Nature et Environnement) in the county of Seine-et-Marne, it was the North American, Paris-based journalist Anita Elash, a correspondent for the US newspaper *The World*, who contacted the collective, warning them that the issue of shale was not only about gas but also about oil (a resource long extracted in the county by conventional methods), and that some North American companies were preparing plans for exploring the Paris Basin. Elash put the county’s activists in touch with their US counterparts, with whom information exchanges could then begin. Marie-Paule Duflot, former chemistry and physics teacher, and activist of the Nature et Environnement 77 Collective. Interview with the author, 13 July 2016 Champs-sur-Marne.

4 The granting of the licenses was in fact publicly documented. The issue was that many stakeholders (including ministers, administrations, and NGOs) were unaware of the granting, since obtaining permission for shale gas exploration does not require a special procedure in France. This is why Chailleux (2016: 520), citing Pepper Culpepper 2010 work, speaks of “quiet politics” to describe the inconspicuousness of the assignment procedure.

5 Odile Autret, former shepherd, nursing assistant, and activist of Ardèche’s anti-shale Collectif 07. Interview with the author, 07 July 2016, Aubenas.

6 Stéphane Milhomme et al., “Total récupère son permis d’exploration de gaz de schiste de Montélimar”, France Bleu, 28 January 2016 (https://www.francebleu.fr/infos/climat-environnement/total-recupere-son-permis-d-exploration-de-gaz-de-schiste-de-montelimar-1453996881, accessed 16 January 2021). In December 2019, Schuepach was paid one million euros for the cancellation of its permit. Pierre-Jean Pluvy and France Bleu Drôme Ardèche, “Un million d’euros pour Schuepbach empêché d’explorer le gaz de schiste en Ardèche”, France Bleu, 19 December 2019 (https://www.francebleu.fr/infos/environnement/un-million-d-euros-pour-schuepbach-empeche-d-explorer-le-gaz-de-schiste-en-ardeche-1576772025, accessed 16 January 2021).

7 However, the argument on the obsolescence of the mining code is challenged by Olivier Appert, former president of the French Petroleum Institute, who argues that “each time there is a change in environmental regulations, it is incorporated into the mining code”. Olivier Appert, interview with the author, 25 August 2016, Rueil-Malmaison.

8 The deficit model attributes public skepticism or hostility to science and technology to a lack of understanding, resulting from a lack of information.

9 Odile Autret, interview with the author, cit.; Gilbert Béal, computer technician and member of the Collective 07. Interview with the author, 07 July 2016.

10 After the passing of the Jacob Bill, some anti-fracking activists voiced their opposition to what they called the “collectives’ dynamics,” judging it too political and partisan. This dissent within the network led to the creation of the association No Fracking France by E. Bourgue and A. Picot (Chailleux 2015: 273).

11 It is also likely that, on the part of the collectives and especially the ones in the southeast, there was a feeling of disempowerment vis-à-vis an association based in Paris.

12 No Fracking France, Scientific Seminar Shale Gas. 25 November 2011. Compte-rendu “No Fracking France”.

13 Balvet, interview with the author, cit.

14 Robert Pilli, coordinator of the Rhône-Alpes Collective “Stop au gaz de schiste” Interview with the author, 25 May 2016, online.

15 Alain Souléliac, former entrepreneur, and member of Collective 07. Interview with the author, 30 June 2016, Aubenas.

16 Association pour la Taxation des Transactions financières et pour l’Action Citoyenne (Association for the Taxation of financial Transactions and Citizen’s Action, ATTAC) is an activist organization originally created to promote the establishment of a tax on foreign exchange transactions.

17 Jacques Cambon, independent hydrogeologist and member of ATTAC France. Interview with the author, 26 May 2016, online.

18 Robert Pili, interview with the author, cit.; Jacques Cambon, interview with the author, cit.

19 However, the report was not followed up because, according to Adéline Mathien, a member of FNE, the government wanted to reframe the shale gas dossier in its own way Adeline Mathien, coordinator of the Energy network, Association France Nature Environnement. Interview with the author, 02 June 2016, Paris.

20 “A slippery decision: Chevron oil pollution in Ecuador”, DW, 09 August 2016 (https://www.dw.com/en/a-slippery-decision-chevron-oil-pollution-in-ecuador/a-18697563, accessed 17 January 2021)

21 Barbara Siegienczuk, advertising agent and ‘Occupy Chevron’ campaign leader. Interview with the author. 27 November 2015, Żurawlów.

22 Teresa Adamska, co-director, Centrum Zrównoważonego Rozwoju. Interview with the author, 30 November 2015, Lodz.

23 Marcin Stoczkiewicz, Head of climate and energy program, ClientEarth Poland. Interview with the author, 02 December 2015. The proposal was scrapped.

24 Nick Meynen, “Fracking: Activists occupy Chevron in Poland”, *ejolt*, 14 June 2013 (www.ejolt.org/2013/06/fracking-activists-occupy-chevron-in-poland/, accessed 17 January 2021).

25 Barbara Siegienczuk, interview with the author, cit.

26 Ewa Pajuro, “Gaz łupkowy na Lubelszczyźnie: Zwolennicy i przeciwnicy rozmawiali o łupkach”, Lubelski Kurier, 8 October 2013 (https://kurierlubelski.pl/gaz-lupkowy-na-lubelszczyznie-zwolennicy-i-przeciwnicy-rozmawiali-o-lupkach/ar/1011691, accessed 16 January 2021).

27 Barbara Siegienczuk, interview with the author, cit.

28 “Gaz de schiste: le gouvernement a désormais les clefs en main pour abroger les permis”. José Bové’ website, 14 September 2011 (http://jose-bove.eu/gaz-de-schiste-le-gouvernement-a?lang=fr, accessed 16 January 2021).

29 Adamska, interview with the author, cit.

30 Jacqueline Balvet, interview with the author, cit.

## References

[CR1] Aczel MR, Makuch KE, Chibane M (2018). How much is enough? Approaches to public participation in shale gas regulation across England, France, and Algeria. The Extractive Industries and Society.

[CR2] Baudrin M, Dauguet B, Deias D, Raimbault B (2014). “On n’est pas des cowboys”. Controversy over the exploitation of shale gases and the strategy of oil industry. Revue d’anthropologie des connaissances.

[CR3] Callon M, Lascoumes P, Barthe Y (2001). Agir dans un monde incertain. Essai sur la démocratie technique.

[CR4] Cantoni R, Craig J, Gerali F, MacAulay F, Sorkhabi R (2018). Second Galicia? Poland’s shale gas rush through historical lenses. “History of the European oil and gas industry”, special publications.

[CR5] Cantoni R, Klaes ML, Lackerbauer S, Foltyn C, Keller R (2018). Shale tales: politics of knowledge and promises in Europe’s shale gas discourses. In Darrick T. Evensen (guest ed.). “Social aspects of unconventional hydrocarbon development globally,” guest edited by, Special issue. The Extractive Industries and Society.

[CR6] Cantoni R, Lis A, Stasik A, Szolucha A (2018). Creating and debating energy citizenship: the case of Poland’s shale gas. Energy, resource extraction and society. Impacts and contested futures.

[CR7] Chailleux S (2015). Non au gaz de schiste ! : cadrages et débordements de la controverse sur les hydrocarbures non conventionnels en France et au Québec.

[CR8] Chailleux S (2016). Incertitude et action publique. Définition des risques, production des savoirs et cadrage des controverses. Rev. Int. Polit. Comp..

[CR9] Chailleux S, Moyson S, Weible CM, Heikkila T, Ingold K, Fischer M (2016). The French ban on hydraulic fracturing and the attempts to reverse it : social mobilization, professional forums, and coalition strategies. Policy debates on hydraulic fracturing. Comparing coalition politics in north america and europe.

[CR10] Chateauraynaud F, Debaz J (2013). Scénariser les possibles énergétiques. Les gaz de schiste dans la matrice des futurs. Mouvements.

[CR11] Cotton M, Rattle I, Van Alstine J (2014). Shale gay policy in the United Kingdom: an argumentative discourse analysis. Energy Policy.

[CR13] Devine-Wright P, Murphy J (2007). Energy citizenship: psychological aspects of evolution in sustainable energy technologies. Governing technologies for sustainability.

[CR14] EIA (2011). World shale gas resources: an initial assessment of 14 regions outside the United States.

[CR15] Epstein S (1995). The construction of lay expertise: AIDS activism and the forging of credibility in the reform of clinical trials. Science, Technology, & Human Values.

[CR53] European Commission (2022). Lubelskie. https://s3platform-legacy.jrc.ec.europa.eu/documents/20182/130357/Backgrounddoc_Lubelskie_EN_%281%29.pdf/ade1834c-c03b-4794-aab3-cde37f08eddc (accessed 12 July 2022).

[CR52] Fleming R (2017). The new German ‘fracking’ package. Journal of Energy & Natural Resources Law.

[CR16] Funtowicz SO, Ravetz JR (1993). Science for the post-normal age. Futures.

[CR17] Geoffron P, Gamper-Rabindran S (2018). France: the power of public opposition—from permits to protests to bans. The shale dilemma: a global perspective on fracking and shale development.

[CR19] Hayes G (2006). Vulnerability and disobedience: new repertoires in French environmental protests. Environmental Politics.

[CR20] Jasanoff S (1995). Science at the bar.

[CR21] Jaspal R, Nerlich B (2013). Fracking in the UK press: threat dynamics in an unfolding debate. Public Understanding of Science.

[CR22] Jaspal R, Nerlich B, Lemańczyk S (2014). Fracking in the Polish press: geopolitics and national identity. Energy Policy.

[CR23] Keeler JTS, Wang Y, Hefley W (2016). The politics of shale gas and anti-fracking movements in France and the UK. The global impact of unconventional shale gas development. Economics, policy, and interdependence.

[CR24] Lis A (2020). Climate and energy politics in Poland: debating carbon dioxide and shale gas.

[CR25] Lis A, Stankiewicz P (2017). Framing shale gas for policy-making in Poland. J. Environ. Pol. Plann..

[CR26] Lis A, Stasik A (2017). Hybrid forums, knowledge deficits and the multiple uncertainties of resource extraction: negotiating the local governance of shale gas in Poland. Energy Research & Social Science.

[CR28] Materka E (2012). End of transition? Expropriation, resource nationalism, fuzzy research, and corruption of environmental institutions in the making of the shale gas revolution in northern Poland. Debatte: Journal of Contemporary Central and Eastern Europe.

[CR29] Materka E (2012). Poland’s quiet revolution: The unfolding of shale gas exploration and its discontents in Pomerania. CEJISS.

[CR30] Mau S (2019). The metric society: on the quantification of the social.

[CR27] Mercado M-T, Álvarez À, Herranz JM (2014). The fracking debate in the media: the role of citizen platforms as sources of information. ESSACHESS—J. Commun. Stud..

[CR31] Metze T (2017). Fracking the debate. Frame shifts and boundary work in Dutch decision making on shale gas. J. Environ. Pol. Plann..

[CR32] Molinatti G, Simonneau L (2015). A socioenvironmental shale gas controversy: scientists’ public communications, social responsibility and collective versus individual positions. Sci. Commun..

[CR33] Porter TM (1995). Trust in numbers. The pursuit of objectivity in science and public life.

[CR34] Rootes CA, Redclift M, Woodgate G (1997). Environmental movements and green parties in western and eastern Europe. The international handbook of environmental sociology.

[CR35] Stankiewicz P (2013). “Razem o łupkach”: czyli jak prowadzić dialog publiczny przy poszukiwaniu i wydobyciu gazu z łupków. Przegląd Geologiczny.

[CR36] Stankiewicz P, Stasik A, Suchomska J (2015). Od informowania do współdecydowania i z powrotem. Prototypowanie technologicznej demokracji. Studia Socjologiczne.

[CR51] Stasik A (2018). Global controversies in local settings: anti-fracking activism in the era of Web 2.0. Journal of Risk Research.

[CR37] Stasik A (2019). Współwytwarzanie wiedzy o technologii. Gaz łupkowy jako wyzwanie dla zbiorowości.

[CR38] Steger T, Milicevic M, Leonard L, Buryn Kedzior S (2014). One global movement, many local voices: discoure(s) of the global anti-fracking movement. Occupy the earth. Global environmental movements.

[CR39] Stoczkiewicz M (2013). Black paper. Implementation of EU climate and energy law in Poland.

[CR40] Sufin-Jacquemart E, Martín-Sosa Rodríguez S (2015). Occupy chevron Poland: squatters and farmers in the same trenches. Global resistance to fracking communities rise up to fight climate crisis and democratic deficit.

[CR44] Szostek M, Jasińska-Kania A (2012). Świadomość ekologiczna polskiego społeczeństwa. Wartości i zmiany. Przemiany postaw Polaków w jednoczącej się Europie.

[CR41] Szołucha A, Whitton J, Cotton M, Charnley-Parry IM, Brasier K (2018). Community understanding of risk from fracking in the UK and Poland: how democracy- and justice-based concerns amplify risk perceptions. Governing shale gas: development, citizen participation and decision making in the US, Canada, Australia and europe.

[CR42] Szołucha A (2018). Energy, resource extraction and society. Impacts and contested futures.

[CR43] Szołucha A (2021). Gaz łupkowy w Polsce. Historia, magia, protest.

[CR45] Terral P-M (2012). La fronde contre le gaz de schiste: essai d’histoire immédiate d’une mobilisation éclair (2010–2011). Ecologie & politique.

[CR46] Terral P-M (2014). Les oppositions au gaz de schiste dans le monde: des protestations nationales à un mouvement citoyen transnational ?. Ecologie & politique.

[CR47] Wahlund M, Palm J (2022). The role of energy democracy and energy citizenship for participatory energy transitions: a comprehensive review. Energy Research & Social Science.

[CR48] Weible CM, Heikkila T, Ingold K, Fischer M (2016). Policy debates on hydraulic fracturing. Comparing coalition politics in north america and europe.

[CR49] Wynne B (1992). Misunderstood misunderstanding: Social identities and public uptake of science. Public Understanding of Science.

[CR50] Wynne B, Lash S, Szerszynski B, Wynne B (1996). May the sheep safely graze? A reflexive view of the expert-lay knowledge divide. Risk, environment and modernity: towards a new ecology.

